# Supplementary material for: Plasmalogen Deficiency and Overactive Fatty Acid Elongation Biomarkers in Serum of Breast Cancer Patients Pre- and Post-Surgery—New Insights on Diagnosis, Risk Assessment, and Disease Mechanisms
Source: Cancers (Basel). 2021 Aug 19;13(16):4170. doi: 10.3390/cancers13164170 (PMC8391794; doi:10.3390/cancers13164170)
Supplement: Supplementary file 1 [file cancers-13-04170-s001.zip › cancers-1256330.suppl.pdf]

Supplementary Materials

Plasmalogen Deficiency and Overactive Fatty Acid Elongation Biomarkers in Serum of Breast Cancer Patients Pre- and Post-Surgery—New Insights on Diagnosis, Risk Assessment, and Disease Mechanisms

Satomi Tomida, Dayan B. Goodenowe, Teruhide Koyama, Etsuko Ozaki, Nagato Kuriyama, Midori Morita, Yasuyo Yamazaki, Koichi Sakaguchi, Ritei Uehara and Tetsuya Taguchi

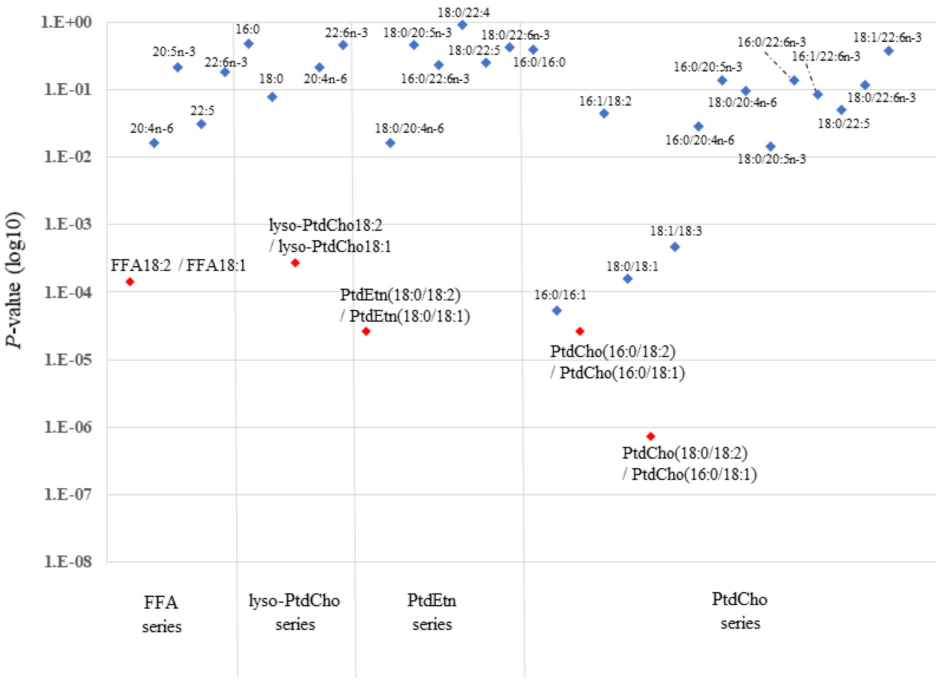

Figure S1. Scatter plots of the *p*-value (log10) based on *t*-test comparison between control and BC on the metabolites of FFA, Lyso-PtdCho, PtdEtn, and PtdCho series.

Table S1. Lipid metabolites observed to have significantly different levels in BC versus control.

**Free Fatty Acids**	**[M-H]⁻**	**Phosphatidylethanolamine**	**[M-H]⁻**	**Phosphatidylcholines**	**[M-H]⁻**
FFA 18:1	281.2486	PtdEtn 18:0/18:1	744.5548	PtdCho 16:0/16:1	732.5537
FFA 18:2	279.2329	PtdEtn 18:0/18:2	742.5392	PtdCho 16:0/16:0	734.5694
FFA 20:4n-6	303.2329	PtdEtn 18:0/20:4n-6	766.5392	PtdCho 16:1/18:2	756.5537
FFA 20:5n-3	301.2173	PtdEtn 18:0/20:5n-3	764.5235	PtdCho 16:0/18:2	758.5694
FFA 22:5	329.2486	PtdEtn 16:0/22:6n-3	762.5079	PtdCho 16:0/18:1	760.5850
FFA 22:6n-3	327.2329	PtdEtn 18:0/22:4	794.5705	PtdCho 16:0/20:5n-3	780.5537
		PtdEtn 18:0/22:5	792.5548	PtdCho 16:0/20:4n-6	782.5694
		PtdEtn 18:0/22:6n-3	790.5392	PtdCho 18:1/18:2	784.5850
Lyso-Phosphatidylcholines	[M]⁺	Ethanolamine Plasmalogens	[M-H]⁻	PtdCho 18:0/18:2	786.6007
lyso-PtdCho 16:0	496.3397	EtnPls 18:0/18:2	726.5443	PtdCho 18:0/18:1	788.6163
lyso-PtdCho 18:0	524.3710	EtnPls 16:0/20:4n-6	722.5130	PtdCho 16:1/22:6n-3	804.5537
lyso-PtdCho 18:1	522.3554	EtnPls 18:0/20:4n-6	750.5443	PtdCho 16:0/22:6n-3	806.5694
lyso-PtdCho 18:2	520.3397	EtnPls 18:0/20:5n-3	748.5286	PtdCho 18:0/20:5n-3	808.5850
lyso-PtdCho 20:4n-6	544.3397	EtnPls 16:0/22:6n-3	746.5130	PtdCho 18:0/20:4n-6	810.6007
lyso-PtdCho 22:6n-3	568.3397	EtnPls 18:0/22:5	776.5599	PtdCho 18:1/22:6n-3	832.5850
		EtnPls 18:0/22:6n-3	774.5443	PtdCho 18:0/22:6n-3	834.6007
		EtnPls 18:1/22:6n-3	772.5286	PtdCho 18:0/22:5	836.6163

M-H means the de-protonated version of the neutral mass: M (neutral) minus a proton [H+] = [M-H]⁻

Table S2. The data of individual species which were observed to have significantly different levels in control versus BC (control means as 1.00 and BC means as relative intensity to control).

**Free Fatty Acids (FFA)**																
	**Mean Intensity Normalized to Respective Control**									***p*-values**						
	**CTL-1**	**BC-1**	**CTL-2**	**BC-2**	**BC-2 (S0)**	**BC-2 (S1)**	**BC-2 (S2)**	**BC-2 (Pre)**	**BC-2 (Post)**	**BC-1**	**BC-2**	BC-2 (S0)	BC-2 (S1)	BC-2 (S2)	BC-2 (Pre)	BC-2 (Post)
FFA 18:1	1.00	0.98	1.00	0.95	0.93	0.94	0.98	0.94	0.97	0.630	0.044	0.118	0.092	0.574	0.098	0.513
FFA 18:2	1.00	0.81	1.00	0.83	0.77	0.80	0.90	0.85	0.96	**0.002**	**<0.001**	**0.004**	**0.001**	0.142	0.010	0.598
FFA 20:4n-6	1.00	0.79	1.00	0.87	0.89	0.87	0.85	0.78	0.88	0.055	0.103	0.453	0.245	0.225	0.034	0.280
FFA 20:5n-3	1.00	0.76	1.00	0.70	0.67	0.73	0.67	0.70	0.71	0.192	0.042	0.267	0.223	0.192	0.149	0.157

FFA 22:5	1.00	0.83	1.00	0.89	0.86	0.92	0.87	0.89	0.97	0.058	0.104	0.295	0.402	0.264	0.235	0.698
FFA 22:6n-3	1.00	0.87	1.00	0.84	0.87	0.83	0.83	0.76	0.68	0.283	0.050	0.421	0.161	0.197	0.036	**0.005**
Lyso-Phosphatidylcholines (lyso-PtdCho)																
	Mean Intensity Normalized to Respective Control									*p*-values						
	CTL-1	BC-1	CTL-2	BC-2	BC-2 (S0)	BC-2 (S1)	BC-2 (S2)	BC-2 (Pre)	BC-2 (Post)	BC-1	BC-2	BC-2 (S0)	BC-2 (S1)	BC-2 (S2)	BC-2 (Pre)	BC-2 (Post)
lyso-PtdCho 16:0	1.00	0.61	1.00	0.73	0.73	0.71	0.76	0.74	0.72	**<0.001**	**<0.001**	**0.001**	**<0.001**	**0.001**	**<0.001**	**<0.001**
lyso-PtdCho 18:0	1.00	0.52	1.00	0.63	0.64	0.63	0.62	0.64	0.68	**<0.001**	**<0.001**	**0.001**	**<0.001**	**<0.001**	**<0.001**	**<0.001**
lyso-PtdCho 18:1	1.00	0.61	1.00	0.79	0.85	0.76	0.80	0.78	0.74	**<0.001**	**<0.001**	0.101	**<0.001**	**0.006**	**<0.001**	**<0.001**
lyso-PtdCho 18:2	1.00	0.52	1.00	0.66	0.65	0.61	0.72	0.67	0.91	**<0.001**	**<0.001**	**0.001**	**<0.001**	**0.002**	**<0.001**	0.234
lyso-PtdCho 20:4n-6	1.00	0.64	1.00	0.87	0.86	0.84	0.94	0.87	0.76	**<0.001**	**0.004**	0.076	**0.007**	0.379	0.017	**<0.001**
lyso-PtdCho 22:6n-3	1.00	0.64	1.00	0.74	0.71	0.74	0.76	0.73	0.85	**<0.001**	**<0.001**	**0.010**	**0.002**	0.010	**<0.001**	0.042
Phosphatidylethanolamine (PtdEtn)																
	Mean Intensity Normalized to Respective Control									*p*-values						
	CTL-1	BC-1	CTL-2	BC-2	BC-2 (S0)	BC-2 (S1)	BC-2 (S2)	BC-2 (Pre)	BC-2 (Post)	BC-1	BC-2	BC-2 (S0)	BC-2 (S1)	BC-2 (S2)	BC-2 (Pre)	BC-2 (Post)
PtdEtn 18:0/18:1	1.00	1.01	1.00	0.96	0.92	0.98	0.96	1.09	1.08	0.868	0.476	0.442	0.827	0.126	0.409	0.653
PtdEtn 18:0/18:2	1.00	0.83	1.00	0.82	0.66	0.76	0.97	0.89	0.94	0.081	0.010	**0.003**	**0.005**	0.830	0.275	0.611
PtdEtn 18:0/20:4n-6	1.00	0.93	1.00	0.86	0.72	0.90	0.89	0.86	0.77	0.565	0.038	0.020	0.266	0.354	0.132	0.020
PtdEtn 18:0/20:5n-3	1.00	1.03	1.00	0.93	0.87	0.98	0.93	0.92	0.75	0.801	0.446	0.435	0.856	0.618	0.506	0.037
PtdEtn 16:0/22:6n-3	1.00	1.00	1.00	0.66	0.56	0.75	0.63	0.63	0.54	0.979	0.011	0.115	0.231	0.107	0.052	0.016
PtdEtn 18:0/22:4	1.00	1.07	1.00	1.04	1.05	1.03	1.03	1.01	0.78	0.445	0.601	0.632	0.690	0.769	0.942	0.019
PtdEtn 18:0/22:5	1.00	0.93	1.00	1.09	1.11	1.11	1.05	1.06	0.98	0.560	0.196	0.352	0.202	0.672	0.505	0.851
PtdEtn 18:0/22:6n-3	1.00	1.05	1.00	0.91	0.87	0.95	0.92	0.89	0.72	0.684	0.263	0.319	0.598	0.517	0.279	**0.008**

Ethanolamine Plasmalogens (PlsEtn)																
	Mean Intensity Normalized to Respective Control									*p*-values						
	CTL-1	BC-1	CTL-2	BC-2	BC-2 (S0)	BC-2 (S1)	BC-2 (S2)	BC-2 (Pre)	BC-2 (Post)	BC-1	BC-2	BC-2 (S0)	BC-2 (S1)	BC-2 (S2)	BC-2 (Pre)	BC-2 (Post)
EtnPls 18:0/18:2	1.00	0.74	1.00	0.76	0.67	0.77	0.79	0.74	0.76	**0.007**	**<0.001**	**0.003**	**0.004**	0.015	**<0.001**	**0.002**
EtnPls 16:0/20:4n-6	1.00	0.74	1.00	0.64	0.67	0.65	0.62	0.61	0.54	0.108	**<0.001**	0.048	**0.004**	**0.006**	**0.001**	**<0.001**
EtnPls 18:0/20:4n-6	1.00	0.64	1.00	0.54	0.53	0.54	0.53	0.51	0.42	0.021	**<0.001**	**0.006**	**<0.001**	**0.001**	**<0.001**	**<0.001**
EtnPls 18:0/20:5n-3	1.00	0.66	1.00	0.50	0.48	0.51	0.49	0.49	0.38	0.033	**<0.001**	**0.003**	**<0.001**	**<0.001**	**<0.001**	**<0.001**
EtnPls 16:0/22:6n-3	1.00	0.74	1.00	0.53	0.48	0.57	0.52	0.54	0.40	0.094	**<0.001**	**0.001**	**<0.001**	**<0.001**	**<0.001**	**<0.001**
EtnPls 18:0/22:5	1.00	0.71	1.00	0.57	0.47	0.60	0.61	0.57	0.64	0.016	**<0.001**	**<0.001**	**<0.001**	**0.002**	**<0.001**	**<0.001**
EtnPls 18:0/22:6n-3	1.00	0.64	1.00	0.52	0.50	0.54	0.53	0.52	0.38	0.021	**<0.001**	**0.002**	**<0.001**	**<0.001**	**<0.001**	**<0.001**
EtnPls 18:1/22:6n-3	1.00	0.67	1.00	0.49	0.45	0.51	0.48	0.49	0.42	0.011	**<0.001**	**<0.001**	**<0.001**	**<0.001**	**<0.001**	**<0.001**

Phosphatidylcholines (PtdCho)																
	Mean Intensity Normalized to Respective Control									*p*-values						
	CTL-1	BC-1	CTL-2	BC-2	BC-2 (S0)	BC-2 (S1)	BC-2 (S2)	BC-2 (Pre)	BC-2 (Post)	BC-1	BC-2	BC-2 (S0)	BC-2 (S1)	BC-2 (S2)	BC-2 (Pre)	BC-2 (Post)
PtdCho 16:0/16:1	1.00	1.41	1.00	1.29	1.19	1.30	1.40	1.24	0.98	**0.001**	**0.001**	0.198	**0.009**	**0.002**	0.026	0.867
PtdCho 16:0/16:0	1.00	1.03	1.00	1.04	0.96	1.10	1.03	1.01	0.93	0.642	0.383	0.641	0.115	0.705	0.842	0.228
PtdCho 16:1/18:2	1.00	1.01	1.00	1.03	0.95	1.04	1.06	1.01	0.98	0.919	0.544	0.564	0.545	0.411	0.800	0.711
PtdCho 16:0/18:2	1.00	0.89	1.00	0.90	0.86	0.87	0.96	0.92	0.95	**0.009**	**0.001**	0.016	**0.003**	0.396	0.040	0.267
PtdCho 16:0/18:1	1.00	1.10	1.00	1.10	1.10	1.09	1.12	1.08	0.96	0.036	**0.007**	0.103	0.071	0.030	0.083	0.398
PtdCho 16:0/20:5n-3	1.00	0.99	1.00	0.87	0.87	0.92	0.82	0.89	1.02	0.919	0.011	0.173	0.307	0.028	0.120	0.766
PtdCho 16:0/20:4n-6	1.00	1.00	1.00	0.95	0.96	0.96	0.96	0.91	0.87	0.950	0.149	0.449	0.386	0.400	0.025	**0.002**
PtdCho 18:1/18:2	1.00	0.94	1.00	0.94	0.96	0.94	0.94	0.94	0.93	0.182	0.065	0.469	0.172	0.208	0.135	0.091

PtdCho 18:0/18:2	1.00	0.84	1.00	0.87	0.84	0.86	0.89	0.89	0.96	**<0.001**	**<0.001**	**0.005**	**0.001**	0.018	**0.006**	0.385
PtdCho 18:0/18:1	1.00	0.93	1.00	0.99	1.01	0.99	0.98	0.98	0.85	0.244	0.755	0.928	0.850	0.743	0.665	0.017
PtdCho 16:1/22:6n-3	1.00	0.94	1.00	0.95	0.93	1.00	0.94	0.92	1.13	0.392	0.452	0.535	0.999	0.517	0.303	0.133
PtdCho 16:0/22:6n-3	1.00	1.02	1.00	0.89	0.93	0.90	0.88	0.85	0.75	0.787	**0.008**	0.338	0.082	0.037	**0.003**	**<0.001**
PtdCho 18:0/20:5n-3	1.00	0.97	1.00	0.89	0.94	0.94	0.81	0.88	0.89	0.602	**0.002**	0.365	0.252	**0.001**	0.013	0.022
PtdCho 18:0/20:4n-6	1.00	1.00	1.00	0.98	1.03	1.00	0.94	0.92	0.85	0.994	0.571	0.703	0.973	0.291	0.123	**0.003**
PtdCho 18:1/22:6n-3	1.00	1.03	1.00	0.95	1.01	0.99	0.89	0.89	0.96	0.608	0.277	0.895	0.872	0.106	0.061	0.548
PtdCho 18:0/22:6n-3	1.00	0.98	1.00	0.88	0.95	0.91	0.81	0.83	0.75	0.800	0.019	0.585	0.226	0.016	0.012	**<0.001**
PtdCho 18:0/22:5	1.00	0.93	1.00	0.88	1.01	0.94	0.76	0.87	0.69	0.380	0.049	0.932	0.452	**0.007**	0.110	**<0.001**

BC; breast cancer, CTL-1; control of study 1, CTL-2; control of study 2, BC-1; BC of study1, BC-2; BC of study 2, S(0); stage 0, S(1); stage 1, S(2); stage 2, (Pre); pre-surgery, (Post); post-surgery
Significant *p*-values (< 0.010) using welch's t test are indicated in bold.
